# Analysis of the relationship between the amplitude of aortic wall motion and heart function

**DOI:** 10.1007/s10396-022-01238-y

**Published:** 2022-07-16

**Authors:** Hideki Tashiro, Samon Koyanagi, Akihiro Honda, Toshikatu Nonaka, Youhei Ohkubo, Chisana Naganuma, Daisuke Fukui, Kenichi Ichimura, Terufumi Sakai

**Affiliations:** 1 Division of Cardiology, St. Mary’s Hospital, 422 Tubuku-honmachi, Kurume, Japan; 2 Hara School of Nursing, 6-40-7 Aoba, Higashi Ward, Fukuoka, Japan; 3 Division of Cardiovascular Medicine, Department of Internal Medicine, Kurume University, 67 Asahi-machi, Kurume, Japan; 4 Clinical Laboratory Center, Saiseikai Omuta Hospital, 810 Takuma, Ohmuta, Japan; 5 Clinical Laboratory Center, Tenjin-Kai Shin-Koga Hospital, 120 Tenjin-machi, Kurume, Japan; 6 Clinical Laboratory Center, Asakura Medical Association Hospital, 422-1 Raiha, Asakura, Japan; 7 Division of Cardiology, Inoue-Kai Sasaguri Hospital, 94 Onaka, Sasaguri, Japan; 8 Clinical Laboratory Center, St. Mary’s Hospital, 422 Tubuku-honmachi, Kurume, Japan; 9 Division of Functional Recovery, St. Mary’s Hospital, 422 Tubuku-honmachi, Kurume, Japan

**Keywords:** Aortic wall motion, M-mode echocardiography, Cardiac function

## Abstract

**Purpose:**

Because the posterior wall of the aorta and left atrium are interlocked, the amplitude of motion of the aortic wall (AMAW) may reflect cardiac and vessel functions. This study examined the relationship between cardiac and vessel functions and AMAW.

**Methods:**

Patients with cardiovascular diseases or patients undergoing health examinations who visited a participating hospital and underwent echocardiography and brachial–ankle pulse-wave velocity (baPWV) examinations were registered. The correlations between echocardiographic indices, ankle–brachial index, and baPWV and AMAW on M-mode echocardiography were analyzed.

**Results:**

Overall, 184 patients were enrolled. Heart rate (*r* =  − 0.1587), ejection fraction (EF; *r* = 0.3240), wall thickness (*r* =  − 0.1598), peak early diastolic mitral annular velocity (E) to peak early diastolic mitral annular velocity ratio (e’; *r* =  − 0.2463), and baPWV (*r* =  − 0.1928) significantly correlated with AMAW. In the stratified multiple regression analysis, E/e’ (standardized partial regression coefficients =  − 0.1863) and mean baPWV (standardized partial regression coefficients =  − 0.1917) in patients with an EF of ≥ 60% (*n* = 114) significantly correlated with AMAW. In patients with an EF of < 60% (*n* = 70), E/e’ (standardized partial regression coefficients =  − 0.2443) significantly correlated with AMAW.

**Conclusion:**

Because E/e’ correlated with AMAW in patients with an EF of < 60% or ≥ 60%, AMAW might be an indicator of left atrial pressure elevation. Moreover, because AMAW correlated with baPWV in patients with an EF of ≥ 60%, changes in the restricted left atrial volume might influence diastolic dysfunction. AMAW may be related to cardiac and vessel functions.

## Introduction

In echocardiography, the M-mode has been the most frequently used modality since its introduction. However, displaying a two-dimensional structure is difficult, because the ultrasound line is fixed. This fact reduces the frequent use of the M-mode. Nevertheless, the M-mode offers an advantage through the high sampling rate, which results in high time resolution [1]. The M-mode allows easy and precise recognition of the movement of the structures on the ultrasound line. Thus, the dimensional change of the left atrium on the M-mode may reflect left atrial function [2, 3]. The aortic root lies adjacent to the left atrium. Because the anterior wall of the left atrium and aortic root are interlocked on the M-mode and the posterior wall motion of the left atrium is almost absent [2], the aortic root motion may closely influence the function of the left atrium. However, these effects have not been reported precisely. The amplitude of motion of the aortic wall (AMAW) may influence left atrial function [2, 3]. However, these hypotheses have not yet been examined. Thus, this study aimed to investigate the correlation between heart and vessel functions and AMAW.

## Materials and methods

This retrospective study comprised 184 patients who underwent echocardiographic examination and whose ankle–brachial index/pulse-wave velocity were measured. These tests were performed within a period of 1 month. This study was conducted in accordance with the Declaration of Helsinki and was approved by the Ethics Committee of St. Mary’s Hospital (K19-0903). The subjects underwent the aforementioned examinations between January 2016 and December 2019 at the member hospitals of the ECL48 group, Fukuoka; the group includes the Kurume University Hospital, St. Mary’s Hospital, and Shin-Koga Hospital in Kurume; Sasaguri Hospital in Kasuya; Asakura Medical Association Hospital in Asakura; and Saiseikai Omuta Hospital in Omuta. Echocardiographic images, including two-dimensional echocardiography, M-mode echocardiography, and Doppler studies, were obtained using standard parasternal and apical views. This study employed an “all comers” design. Patients with cardiovascular disease or individuals coming for a health examination who visited a participating hospital and underwent echocardiography and baPWV examinations were registered.

**Fig. 1:**
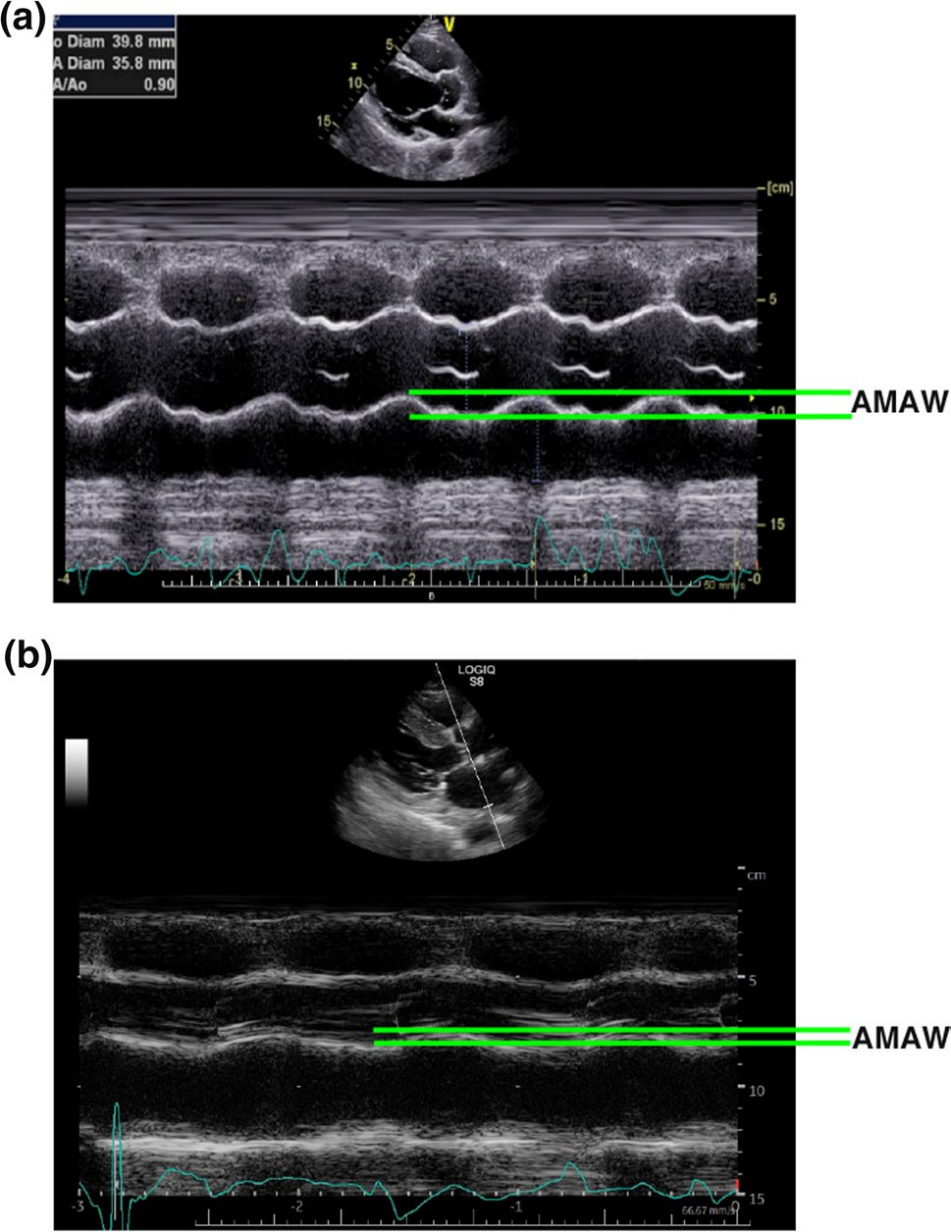
The amplitude of the posterior aortic wall was measured as the amplitude of motion of the posterior aortic wall at the aortic valve level on M-mode echocardiography in the parasternal long-axis view. **a** A male patient with cardiomyopathy in his 30 s. **b** A female patient with heart failure with preserved ejection fraction in her 90 s. AMAW varies across people. Abbreviation: *AMAW* amplitude of motion of the aortic wall

During the conventional two-dimensional echocardiographic examination, left ventricular end-diastolic and end-systolic volumes were measured using the biplane modified Simpson method, and the ejection fraction (EF) was calculated. M-mode echocardiography was performed to measure the interventricular septal thickness, posterior wall thickness, maximum and minimum left atrial diameters, aortic diameter, end-diastolic left ventricular diameter, and AMAW (Fig. 1). The ratio of the maximum and minimum left atrial diameters [4] was calculated. Using the parasternal long-axis view, AMAW was measured as the amplitude of the posterior aortic wall at the aortic valve level in the M-mode (Fig. 1). By applying a conventional pulsed wave Doppler study in the apical view, the transmitral inflow and left ventricular outflow velocities were recorded. The peak velocities of early diastolic (E) and late diastolic (A) inflows were measured, and the E/A ratio was calculated [5]. Using tissue Doppler imaging with the sample volume placed at the basal septal wall in an apical four-chamber view [5], the peak early diastolic mitral annular velocity (e’) was recorded. The brachial–ankle pulse-wave velocity (baPWV) and ankle–brachial index were determined using the Form (Colin, Komaki, Japan) or VaSera (Fukuda Denshi, Tokyo, Japan) system. As the VaSera system cannot directly measure baPWV, it was calculated as follows: baPWV = (0.5934 × body height (cm) + 14.4014) / the time of pulse-wave delay between the arm and ankle [6]. The intraclass correlation coefficients of AMAW were calculated using the values measured by two experienced sonographers in 15 patients.

All variables are expressed as means ± standard deviations. Univariate regression analyses were performed to compare the echocardiographic indices, Doppler indices, baPWV, and ankle–brachial index. Multiple regression analyses were performed using the indices that significantly correlated with AMAW in the stratified univariate analyses. Data analyses were performed using JMP Pro (version 15.1.0; SAS Institute, Cary, USA). Statistical significance was set at *p* < 0.05 using bilateral test.

## Results

Patient characteristics are summarized in Table 1. There were six patients with paroxysmal atrial fibrillation who were also examined during sinus rhythm. Table 2 presents the echocardiographic and Doppler indices, baPWV, and ankle–brachial index. Heart rate (*r* =  − 0.1587), EF (*r* = 0.3240), wall thickness (*r* =  − 0.1598), E/e’ (*r* =  − 0.2463), and baPWV (*r* =  − 0.1928) significantly correlated with AMAW (Table 3). Age, height. body weight, systolic and diastolic blood pressures, end-diastolic left ventricular and aortic diameters, maximum and minimum left atrial diameters, ratio of the maximum and minimum left atrial diameters, E/A, and ankle–brachial index did not correlate with AMAW (Table 3). When stratified analysis of patients with an EF of ≥ 60% and < 60% was performed, the mean baPWV (*r* =  − 0.20459) and E/e’ (*r* =  − 0.19403) significantly correlated with AMAW in patients with an EF of ≥ 60%, and E/e’ (*r* =  − 0.28611) and wall thickness (*r* =  − 0.26258) significantly correlated with AMAW in patients with an EF of < 60% (Table 4). Multiple regression analysis results indicated that in patients with an EF of ≥ 60%, E/e’ (standardized partial regression coefficients =  − 0.1863) and baPWV (standardized partial regression coefficients =  − 0.1917) significantly correlated with AMAW, whereas in patients with an EF of < 60%, only E/e’ (standardized partial regression coefficients =  − 0.2443) significantly correlated with AMAW (Table 5). However, wall thickness did not correlate with AMAW. The intraclass correlation coefficient of AMAW (1,3) and (2,3) was 0.9773 and 0.9450, respectively.

**Table 1:** Background characteristics of patients

Characteristic	Mean ± SD	Characteristic	Patients, *n* (%)
Age (years)	70 ± 12	Male	116 (63)
Height (cm)	161 ± 9	Valvular heart disease	10 (5)
Body weight (kg)	61 ± 13	Old myocardial infarction	29 (16)
Systolic BP (mmHg)	138 ± 19	Angina	32 (17)
Diastolic BP (mmHg)	77 ± 13	Hypertension	115 (63)
Heart rate (beats/min)	70 ± 13	Diabetic mellitus	100 (54)
		Dyslipidemia	110 (60)
		Brain infarction	11 (6)
		Cardiomyopathy	6 (3)
		Arrhythmia	11 (6)
		Aortic disease	3 (2)
		Symptomatic heart failure	29 (16)

*BP* blood pressure, *SD* standard deviation

**Table 2:** Echocardiographic and Doppler indices, pulse-wave velocities, and ankle-brachial index

Variable	Mean ± SD
AMAW (mm)	8.6 ± 2.5
EF (%)	63.6 ± 11.2
LVDd (mm)	44.3 ± 5.7
AoD (mm)	28.4 ± 3.5
maxLAD (mm)	36.0 ± 6.0
minLAD (mm)	29.2 ± 6.7
minLAD/maxLAD	0.77 ± 0.1
Wall thickness (mm)	20.7 ± 3.3
E/A	0.88 ± 0.9
E/e’	11.3 ± 4.6
Mean baPWV (cm/sec)	1700.5 ± 363.1
Mean ABI	1.1 ± 0.1

*A* peak velocity of late diastolic flow, *AMAW* amplitude of the motion of the aorta, *ABI* ankle-brachial index, *AoD* aortic diameter, *baPWV* brachial-ankle pulse-wave velocity, e’, peak early diastolic mitral annular velocity, *E* peak velocity of early diastolic flow, *EF* ejection fraction, *LVDd* end-diastolic left ventricular diameter, *maxLAD* maximum left atrial diameter, *minLAD* minimum left atrial diameter, *SD* standard deviation

**Table 3:** Correlation between AMAW and background, echocardiographic, and Doppler characteristics; baPWV; and ABI

Characteristic	*r*	*p* value
Age	− 0.1321	0.0739
Height	− 0.0263	0.7230
Body weight	0.0565	0.4462
Systolic BP	0.0587	0.4301
Diastolic BP	0.0775	0.2972
Heart rate	− 0.1587	0.0314
LVDd	− 0.0779	0.2931
EF	0.3240	< 0.0001
AoD	0.0391	0.5983
maxLAD	− 0.0476	0.5212
minLAD	− 0.0609	0.5053
minLAD/maxLAD	0.1536	0.0911
Wall thickness	− 0.1598	0.0302
E/A	− 0.0703	0.3447s
E/e’	− 0.2463	0.0007
baPWV	− 0.1928	0.0087
ABI	0.0486	0.5120

*A* peak velocity of late diastolic flow, *AMAW* amplitude of motion of aorta, *ABI* ankle-brachial index, *AoD* aortic diameter, *baPWV* brachial-ankle pulse-wave velocity, e’, peak early diastolic mitral annular velocity, *E* peak velocity of early diastolic flow, *EF* ejection fraction, *LVDd* end-diastolic left ventricular diameter, *maxLAD* maximum left atrial diameter, *minLAD* minimum left atrial diameter

**Table 4:** Stratified analysis of the correlation between AMAW and E/e’, mean PWV, and wall thickness in patients with EF of above and below 60%

Variable	EF ≥ 60% (*n* = 114)		EF < 60% (*n* = 70)	
	*r*	*p*-value	*r*	*p*-value
Mean baPWV	− 0.20459	0.0290	− 0.14925	0.2175
E/e’	− 0.19403	0.0386	− 0.28611	0.0163
Wall thickness	− 0.09364	0.3217	− 0.26258	0.0281

*AMAW* amplitude of motion of aorta, e’, peak early diastolic mitral annular velocity, *E* peak velocity of early diastolic flow, *EF* ejection fraction, *PWV* pulse-wave velocity

**Table 5:** Stratified multiple regression analysis

Variable	Standardized partial regression coefficients	*p*-value
EF ≥ 60%		
E/e’	− 0.1863	0.0436
baPWV	− 0.1917	0.0328
EF < 60%		
E/e’	− 0.2443	0.0396
Wall thickness	− 0.2151	0.0691

*baPWV* brachial-ankle pulse-wave velocity, e’, peak early diastolic mitral annular velocity, *E* peak velocity of early diastolic flow, *EF* ejection fraction

## Discussion

The motion of the aortic wall on M-mode is a well-known phenomenon [1]. Feigenbaum has reported that changes in the left atrial volume are best reflected by the motion of the anterior aortic wall, which is the same as that of the posterior aortic wall [1]. Pratt et al. have reported that the amplitude of motion of the posterior aortic wall correlates with the stroke volume [7]. Additionally, Aizawa et al. have reported that the mean velocity of the motion of the anterior aortic wall during systole on M-mode is related to the stroke volume [8]. However, because this phenomenon is observed on M-mode, almost all cardiologists have lost interest.

Contrastingly, M-mode echocardiography is a fundamental function and is included in almost all devices. Therefore, AMAW can be measured not only with new devices but also with older devices. AMAW might be one of the most easily measurable echocardiographic indices.

AMAW influences left atrial function, because the posterior wall of the aortic root and left atrium are interlocked. Therefore, AMAW might be related to both atrial and vessel functions. In the univariate analysis, heart rate, EF, wall thickness, E/e’, and baPWV significantly correlated with AMAW. EF might be the source of power for aortic movement as it is the index of motion. Pratt et al. suggested that left ventricular ejection contributes to the aortic root motion [7]. Stratified analysis was performed with an EF of ≥ 60% or < 60% because EF was the highest coefficient of determination. In multivariate analyses, only E/e’ significantly correlated with AMAW in patients with an EF of ≥ 60% or < 60%. Ommen et al. reported that E/e’ correlated with the mean left ventricular diastolic pressure, which was used as a surrogate for the mean left atrial pressure [9]. Because AMAW in the patients with an EF of < 60% correlated with the wall thickness and E/e’, AMAW might correlate with the increase in the mean left atrial pressure due to systolic and diastolic dysfunctions. Hsiao et al. reported a correlation between left atrial distensibility and left ventricular filling pressure [10]. Because E/e’ correlated with AMAW in patients with an EF of ≥ 60%, AMAW might be related to the increase in the mean left atrial pressure due to diastolic dysfunction.

The measure of systemic arterial stiffness, known as baPWV, is measured using brachial and tibial arterial wave analyses [11]. Aortic stiffening increases cardiac workload, which leads to left ventricular hypertrophy and heart failure [11]. In this study, AMAW correlated with baPWV in patients with an EF of ≥ 60%. Kishimoto et al. reported that baPWV in patients with heart failure with preserved EF (HFpEF) increased [12]. Melenovsky et al. reported that HFpEF increased the left atrial stiffness [13]. As the aorta encounters the left atrium, an atherosclerotic aorta might restrict the changes in the left atrial volume and increase the mean left atrial pressure. In contrast, AMAW did not correlate with baPWV in patients with an EF of < 60%. Because EF is the source of power for aortic root movement, vessel function may not reflect AMAW in patients with low EF.

Although decreased AMAW might be directly correlated with atherosclerosis, reduced endothelial function and restriction of the left atrium may decrease left ventricular diastolic function.

The major limitations of this study were its retrospective design and small sample size. In this study, stratified multiple regression analyses were performed to investigate the relationship between AMAW and various indices of cardiac and vessel functions. For these analyses, a wide range of cardiac and vessel functions and a large number of subjects for investigation were considered necessary. Therefore, patients with heart failure as well as those who came only for health check-ups were included in this study. However, the sample size may have been insufficient. Additionally, the echocardiographic data were limited to general indices given the retrospective nature of this study. Therefore, despite being important factors, the left atrial volume and left atrial emptying fraction could not be analyzed. Future research should include patients with heart failure in particular.

## Conclusion

AMAW on M-mode correlated with EF, baPWV, and E/e’. Thus, AMAW may be defined based on vascular status and cardiac function. Because the aortic root restricts the left atrium, AMAW may influence left atrial function. Furthermore, AMAW might be one of the indicators of left atrial function.

## References

[1] Instrumentation FH, Feigenbaum H (1994). Echocardiography.

[2] Strunk BL, Fitzgerald JW, Lipton M (1974). The posterior aortic wall echocardiogram. Its relationship to left atrial volume change. Circulation.

[3] Santos ABS, Roca GQ, Claggett B (2016). Prognostic relevance of left atrial dysfunction in heart failure with preserved ejection fraction. Circ Heart Fail.

[4] Koyanagi S, Anan T, Koiwaya Y (1980). The change in the left atrial dimension during diastole. Echocardiographic assessment of mitral stenosis. Jpn Heart J.

[5] Kasner M, Westermann D, Steendijk P (2007). Utility of Doppler echocardiography and tissue Doppler imaging in the estimation of diastolic function in heart failure with normal ejection fraction: a comparative Doppler-conductance catheterization study. Circulation.

[6] Yamashina A, Tomiyama H, Takeda K (2002). Validity, reproducibility, and clinical significance of noninvasive brachial-ankle pulse wave velocity measurement. Hypertens Res.

[7] Pratt RC, Parisi AF, Harrington JJ (1976). The influence of left ventricular stroke volume on aortic motion: an echocardiographic study. Circulation.

[8] Aizawa Y, Hosokawa O, Shibata A (1980). Stroke volume estimated from the aortic root motion in M-mode. Echocardiography. Iyoudenshi to Seitaikougaku.

[9] Ommen SR, Nishimura RA, Appleton CP (2000). Clinical utility of Doppler echocardiography and tissue Doppler imaging in the estimation of left ventricular filling pressures: a comparative simultaneous Doppler-catheterization study. Circulation.

[10] Hsiao SH, Chiou KR, Lin KL (2011). Left atrial distensibility and E/e’ for estimating left ventricular filling pressure in patients with stable angina–a comparative echocardiography and catheterization study. Circ J.

[11] Munakata M (2014). Brachial-ankle pulse wave velocity in the measurement of arterial stiffness: Recent evidence and clinical applications. Curr Hypertens Revi.

[12] Kishimoto S, Kajikawa M, Maruhashi T (2017). Endothelial dysfunction and abnormal vascular structure are simultaneously present in patients with heart failure with preserved ejection fraction. Int J Cardiol.

[13] Melenovsky V, Hwang SJ, Redfield MM (2015). Left atrial remodeling and function in advanced heart failure with preserved or reduced ejection fraction. Circ Heart Fail.

